# Maple Syrup Urine Disease Masquerading as Urea Cycle Disorder: A Tale of Two Clinical Mimics

**DOI:** 10.7759/cureus.9706

**Published:** 2020-08-12

**Authors:** Shahzad Rauf, Talal Almas, Irfan Ullah, Norina Usman, Muhammad Irfan

**Affiliations:** 1 Pediatrics, Khyber Teaching Hospital, Peshawar, PAK; 2 Internal Medicine, Royal College of Surgeons in Ireland, Dublin, IRL; 3 Internal Medicine, Kabir Medical College, Peshawar, PAK; 4 Internal Medicine, Naseer Teaching Hospital, Peshawar, PAK; 5 Internal Medicine, Veterans Affairs Palo Alto Health Care System-Stanford University School of Medicine, Palo Alto, USA; 6 Internal Medicine, Hayatabad Medical Complex, Peshawar, PAK

**Keywords:** maple syrup urine disease, urea cycle disorder, inborn errors of metabolism

## Abstract

Maple syrup urine disease, an inherited disorder of metabolism, is characterised by deficient activity of the branched-chain alpha-keto acid dehydrogenase complex (BCKAD) enzyme, resulting in an accumulation of branched-chain amino acids. While it is classically diagnosed by the means of a neonatal screening panel, it can sometimes remain undetected. In such cases, maple syrup urine disease is noted to elicit a constellation of clinical symptoms characterised by a plethora of neurological and respiratory impairments. A prompt diagnosis and management of the disease therefore remains imperative. Due to the remarkable semblance in the clinical symptoms elicited by maple syrup urine disease and urea cycle disorders, both the ailments should be considered in the list of differential diagnosis in patients presenting with elevated serum ammonia levels in the context of the overarching clinical picture. We chronicle the case of a 25-day-old neonate who presented with unabated seizures. An initial diagnosis of a urea cycle disorder was suspected; however, further diagnostic workup divulged an underlying diagnosis of maple syrup urine disease.

## Introduction

Maple syrup urine disease (MSUD) is a rare autosomal recessive condition in which the metabolism of branched-chain amino acids (BCAAs) is severely impaired, resulting in a pathological buildup of BCAAs [1]. Pathologically increased accumulation of leucine in the brain and blood leads to metabolic decompensation [1]. The prevalence rate of MSUD hovers around 1 in 185,000 people globally, rendering it an exceedingly rare entity [1]. The clinical constellation of symptoms elicited by MSUD is categorized by encephalopathy, neurological developmental delays, and a peculiar maple syrup odor to the urine [1,2]. Upon further investigations, patients with MSUD are noted to have increased levels of branched ketoacids in the urine and elevated BCAA levels in the plasma [1]. In typical manifestations of the ailment, less than 2% of the normal branched-chain alpha-keto acid dehydrogenase complex (BCKAD) activity is noted [1]. Pathologically low levels of the aforesaid enzyme culminate in a concoction of clinical symptoms, including respiratory distress, stupor, cerebral edema, and a multitude of associated neurological and respiratory manifestations [2]. We delineate an interesting case of MSUD in a 25-day-old infant who presented with a history of unabated seizures despite the uptake of anticonvulsants, alluding to the diagnosis of an underlying metabolic disorder.

## Case presentation

We hereby elucidate the case of a 25-day-old male infant who presented to us with complaints of uncontrolled fits despite the uptake of anticonvulsants, including phenobarbitone and phenytoin, and intravenous antibiotics in order to combat his ostensible meningitis. According to the infant’s mother, she witnessed seizures for the first time on the third day of the neonate’s life. The first episode was reported to be three minutes in duration, and consisted of neck retraction, fisting and moaning sounds. The baby was afebrile during and after the episode. The infant had been breastfed since birth. Pertinently, his birth history was uneventful, with no prenatal complications. Interestingly, the baby was a product of a consanguineous marriage, with the mother having undergone one abortion previously at three months of gestation. Thereafter, the patient was admitted to our hospital. The initial impression deduced was that of meningitis; subsequent workup and treatment in line with this diagnosis were thus initiated. After a course of intravenous antibiotics for five days, the infant’s condition remained unaltered. As part of our workup, laboratory tests consisting of a complete blood count, blood culture, basic metabolic panel, and urinalysis were conducted on four separate days. The results obtained from these evaluations are delineated in Table 1. 

**Table 1:** The results obtained from the complete blood count

Parameter	Normal Range	14^th^ May	15^th^ May	18^th^ May	29^th^ May
White Blood Cell Count (WBC)	4-11 (×10^3^)/µL	9.1	8.9	6.9	7.29
Red Blood Cell Count (RBC)	4-6 (x10^6^)/µL	6.15	6.14	6.02	5.54
Hemoglobin	11.5-17.5 g/dL	18.9	20	18.1	15.39
Hematocrit	36-54%	53.7	54.6	54.6	44.61
Mean Corpuscular Volume	76-96 fL	87.3	89	90.6	80.59
Mean Corpuscular Hemoglobin	27-33 pg	30.8	32.6	30.1	27.81
Mean Corpuscular Hemoglobin Concentration	33-35 g/dL	35.3	36.6	33.2	34.51
Platelets	150-450 (×10^3^)/µL	336	165	145	235.3
Neutrophils	40-75%	66.4	58	47.2	45.73
Lymphocytes	20-45%	24.4	32.6	28.5	39.98
Monocytes	2-10%	-	-	-	13.53
Mixed Cell Count	5-20%	9.2	9.4	24.3	-

**Figure 1:**
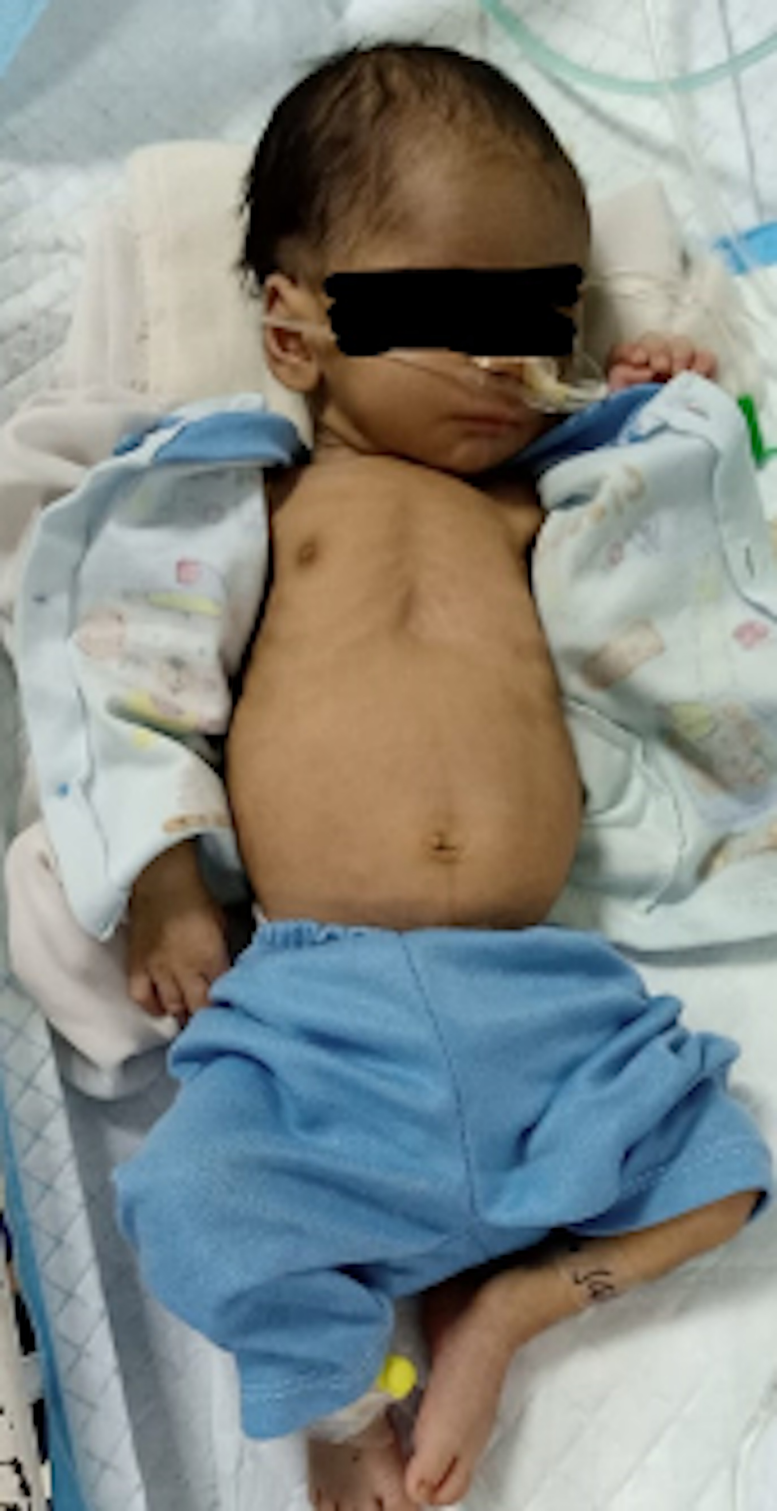
A neonate in visible respiratory distress as elucidated by the recession of his chest wall

By this time, besides sepsis, a possible inborn error of metabolism, was also considered in the list of the differential diagnoses. Subsequent metabolic and septic workup reports were normal except the arterial blood gases (ABGs), which divulged respiratory alkalosis (pH = 7.53) and a decreased blood urea nitrogen value of 6.3 mg/dL (Normal range = 7-20 mg/dL). This was in line with the physical examination findings, which revealed a neonate under obvious respiratory distress (Figure 1).

Interestingly, an exorbitantly elevated serum ammonia level of 722 µmol/L was noted, insinuating an underlying diagnosis of an inborn error of metabolism. A plasma aminogram was thus ordered to further ascertain the underlying etiology. The impression following the protocol described above was that of a urea cycle disorder, leading to the aforementioned clinical manifestations. However, an analysis of serum BCAAs revealed an increased leucine level of 854 μM (normal range = 75-165 μM) and an increased serum valine level of 443 μM (normal range = 126-220 μM), thereby favouring a diagnosis of MSUD. Given that the patient’s serum ammonia level was greater than 500 µmol/L, haemodialysis should have ideally been performed; however, our setup lacked this facility. The patient was therefore initiated on sodium benzoate, an ammonia scavenger. Thereafter, his serum ammonia levels dropped rapidly, and his clinical symptoms abated promptly.

## Discussion

Maple syrup urine disease (MSUD) is a rare inborn error of metabolism that ensues due to defective branched-chain alpha-keto acid dehydrogenase (BCKAD) activity [3]. Its prevalence is reported to hover around 1 in 185,000 people globally [1]. As a result of an autosomal recessive mutation afflicting the genes responsible for encoding BCKAD, persistently elevated serum levels of branched-chain amino acids (BCAAs) are observed [3]. Over time, persistently elevated levels of BCAAs, including leucine, can culminate in metabolic dysfunction [3]. Clinically, patients afflicted with MSUD are noted to manifest a myriad of symptoms including seizures, respiratory distress, brain edema, and potentially death [3]. In patients with MSUD, a peculiar finding of a maple scent emanating from the urine can often prompt suspicions of the disorder [3]. While MSUD commonly presents in neonates, it can also, in rare instances, present in adults [3,4]. In adults, exorbitantly elevated BCAA levels can result in nausea, vomiting, abdominal pain, and neurological affections such as ataxia [4]. Interestingly, the neurological findings elicited by MSUD in the adult population are noted to closely mimic the findings ubiquitous in Wernicke encephalopathy, which can often obscure a timely diagnosis of MSUD [5].

MSUD is classically staged with respect to the existent levels of residual BCKAD enzymatic activity. The classical subset of MSUD that presents in neonates is noted to boast a residual enzymatic activity merely 2% of the normal levels [2]. Due to the severe enzymatic deficiency, grave neurological sequelae are noted in this population [2]. If not managed promptly, this subdivision of MSUD can rapidly progress to lethargy, coma, and even death secondary to brain edema [6]. The intermediate type of the ailment is defined by a residual enzymatic activity 30% of the normal levels [6,7]. Consequently, patients afflicted with this subtype may progress through the neonatal period unfazed; however, over time, these patients experience intellectual impairments, feeding problems, and a slate of symptoms quite similar to those observed in the classical subtype [6]. The intermittent subtype of MSUD often portends the most favourable prognosis. Typically, patients with this subtype often remain unaffected and do not experience the sequelae elicited by the other forms of MSUD [7]. Nevertheless, even patients within this subset can experience neurological deterioration, lethargy, lack of appetite, and nausea in the aftermath of persistent stressors or a poor diet [7].

In our case, our patient presented to us with a history of recurrent, unabated seizures. Considering the standard protocols, an initial diagnosis of neonatal meningitis was suspected, and the neonate was thus commenced on a standard antibiotic regimen. However, our patient’s non-responsiveness to the aforesaid antibiotic regimen, along with unremarkable CBC findings, precluded the diagnosis of meningitis. Thereafter, serum ammonia levels were noted to be exorbitantly elevated, pointing towards an ostensible urea cycle disorder, which results in an exceedingly similar clinical picture [7]. However, a subsequent aminogram, coupled with markedly elevated BCAA levels, favoured a diagnosis of MSUD. There is thus a need for MSUD to be considered in the list of differential diagnoses in a patient suspected to have a urea cycle disorder. Furthermore, prompt diagnosis and management of MSUD remain pivotal in thwarting the grave sequelae that the disease can elicit [8].

## Conclusions

Maple syrup urine disease is a rare autosomal recessive inborn error of metabolism that culminates in defective protein metabolism, leading to pathologically high levels of branched-chain amino acids. Exorbitantly elevated levels of branched-chain amino acids can eventually herald the onset of a multitude of neurological, respiratory, and gastrointestinal symptoms. There is thus an overarching need to promptly diagnose and manage the ailment. Furthermore, maple syrup urine disease can often present with a clinical picture exceedingly similar to that elicited by urea cycle disorders; maple syrup urine disease should, therefore, be considered a differential diagnosis in patients suspected to have an underlying urea cycle disorder.
